# Effects of drylot dimension and group size on growth, physiological, and puberty responses of replacement beef heifers

**DOI:** 10.1093/tas/txag097

**Published:** 2026-07-08

**Authors:** Camila P Prado, Reinaldo F Cooke, Shea J Mackey, Autumn T Pickett, Guilherme A Monteiro, Izadora S de Souza, Courtney L Daigle, Kelsey M Harvey

**Affiliations:** Department of Animal Science, Texas A&M University, College Station, TX 77843, United States; Department of Animal Science, Texas A&M University, College Station, TX 77843, United States; Department of Animal Science, Texas A&M University, College Station, TX 77843, United States; Department of Animal Science, Texas A&M University, College Station, TX 77843, United States; Department of Animal Science, Texas A&M University, College Station, TX 77843, United States; Department of Animal Science, Texas A&M University, College Station, TX 77843, United States; Department of Animal Science, Texas A&M University, College Station, TX 77843, United States; Prairie Research Unit—Mississippi State University, Prairie, MS 39756, United States

**Keywords:** beef heifers, drylot configuration, puberty, stocking density, stress

## Abstract

This experiment evaluated growth, physiological responses, and puberty attainment in beef heifers reared in different stocking densities. Heifers (*n* = 240; 75% Angus × 25% Brahman) were ranked by age (270 ± 1 d), body weight (**BW** on d -3; 232 ± 2 kg) and temperament score on d 0, and assigned to (a) 1 of 6 drylot pens (10 × 14 m pens; 10 heifers/pen) with a stocking density of 14 m^2^/heifer (**HDENS**), (b) 1 of 6 drylot pens (10 × 28 m pens; 20 heifers/pen) with a stocking density of 14 m^2^/heifer (**HDENS-XL**), or (c) 1 of 6 pastures (1.38-ha pastures; 10 heifers/pasture) with a stocking density of 1,380 m^2^/heifer (**CON**). Shrunk BW was recorded (16 h of feed and water withdrawal) on d -3 and 171 to calculate BW gain. Heifers were fitted with an ear tag on d 0 to record behavioral responses. Blood samples were collected weekly for plasma progesterone analysis. Whole blood samples were collected on d 0, 58, 114, and 170 for mRNA isolation. Hair samples from the tail switch were collected on d 0, 30, 58, 85, 114, 142, and 170. Data were analyzed with pen or pasture as experimental unit. No treatment effects were detected (*P* = 0.67) for BW gain (∼0.635 kg/d). Time spent eating was greater (*P* < 0.01) in CON compared with HDENS and HDENS-XL. Time spent non-active, active and highly active were less (*P* < 0.01) in CON compared with HDENS and HDENS-XL. Time spent active was also less (*P* = 0.04) in HDENS compared with HDENS-XL. Hair cortisol concentrations were greater (*P* ≤ 0.05) for HDENS and HDENS-XL compared with CON on d 30 and 170, greater (*P* ≤ 0.04) for HDENS compared with CON and HDENS-XL on d 58, and greater (*P* = 0.03) for HDENS compared with CON on d 142. Expression of *heat shock protein* (**HSP**)-70 mRNA was greater (*P* < 0.01) for HDENS compared with HDENS-XL on d 114, greater (*P* ≤ 0.05) for HDENS compared with HDENS-XL and CON on d 170, and also greater (*P* = 0.03) for HDENS-XL compared with CON on d 170. Expression of HSP-72 mRNA was greater (*P* < 0.01) for HDENS compared with HDENS-XL and CON on d 170. Puberty rate was greater (*P* ≤ 0.05) in CON compared with HDENS in weeks 8, 9, 17 and 23, greater (*P* ≤ 0.05) for CON compared with HDENS and HDENS-XL from weeks 10 to 12, and greater (*P* ≤ 0.05) in CON and HDENS-XL compared with HDENS from weeks 13 to 16 and weeks 18 to 22. Collectively, increasing drylot dimension and group size attenuated physiological stress responses and improved reproductive development of beef heifers reared at a high stocking density.

## Teaser

Beef heifers reared in drylots with high stocking density (14 m^2^/heifer) experience less physical activity, increased chronic stress responses, and delayed puberty compared with cohorts reared on pasture. This experiment evaluated if increasing the size of the drylot, but maintaining the same stocking density (14 m^2^/heifer), would provide more space for physical activity, alleviate stress responses, and hasten reproductive development of beef heifers reared in drylots.

## Introduction

The U.S. beef industry continues to balance productivity with cattle welfare and efficient use of natural resources (FAO 2009). Stocking density is a key management decision influencing these objectives; yet, its implications are often overlooked in cow–calf systems (Asem-Hiablie et al. 2017). Previous work from our group demonstrated that rearing heifers in drylots with high stocking density (14 m^2^/heifer) elicited chronic stress responses associated with delayed puberty attainment compared with heifers reared on pasture (Schubach et al. 2017; Harvey et al. 2024). Given that stress impairs reproductive development (Dobson and Smith 2000), and high stocking density is a recognized stressor for cattle (Huzzey et al. 2012), research is warranted to refine management decisions for intensive heifer development programs (Tucker et al. 2015).

In our companion manuscript, Prado et al. (2026) reported that reducing drylot stocking density from 14 m^2^/heifer to 28 and 42 m^2^/heifer attenuated chronic and cellular stress responses, but did not prevent the delay in puberty attainment compared with heifers on pasture (1,380 m^2^/heifer). Strategies beyond stocking density reduction should be investigated to improve heifer welfare and reproductive outcomes reared in drylot systems. In other livestock systems including dairy cattle, increasing drylot dimensions without changing stocking density increases the total space available for physical activity (Hoerl et al. 2008; Telezhenko et al. 2012; Abdelfattah et al. 2013). Increased drylot dimensions also accommodate larger group sizes, which can promote physical activity (Færevik et al. 2007) and mitigate social stressors (Takeda et al. 2003; Færevik et al. 2007).

To our knowledge, no research has evaluated different combinations of drylot dimensions and group sizes in beef heifers in drylots with high stocking density. Therefore, we hypothesized that increasing drylot and group size will alleviate the detrimental effects of high stocking density on welfare and reproductive development of beef heifers. To test this hypothesis, this experiment compared behavioral, physiological, and reproductive responses in beef heifers reared in drylots with high stocking density (14 m^2^/heifer), but different drylot dimensions and group sizes (10 heifers in 140 m^2^ or 20 heifers in 280 m^2^). As in the companion paper (Prado et al. 2026), heifers reared on pasture were included as positive control (Schubach et al. 2017; Harvey et al. 2024).

## Materials and methods

This experiment was conducted over 2 consecutive years (year 1: November 2023 to April 2024; year 2: November 2024 to April 2025) at the Texas A&M—McGregor Research Center (McGregor, TX), concurrently with the experiment described by Prado et al. (2026). Heifers were cared for in accordance with acceptable practices and experimental protocols approved by the Texas A&M AgriLife Research, Agriculture Animal Care and Use Committee (#2023-018A).

### Animals and treatments

A total of 240 heifers (75% Angus × 25% Brahman) born at the Texas A&M—McGregor Research Center (McGregor, TX) were used in this experiment (120 heifers/year; d 0 to 170) as described by Prado et al. (2026). Heifers were maintained on pasture from birth until weaning (3 weeks prior to the beginning of the experiment), and then maintained in a single 10-ha pasture with ad libitum access to bermudagrass hay (*Cynodon dactylon*), water, and a commercial mineral and vitamin mix. Heifers were ranked by age (270 ± 1 d), shrunk body weight (**BW** on d -3; 232 ± 2 kg), and temperament score (Cooke 2014) on d 0, and randomly assigned to (a) 1 of 6 drylot pens (10 × 14 m pens; 10 heifers/pen) with a stocking density of 14 m^2^/heifer (**HDENS**), (b) 1 of 6 drylot pens (10 × 28 m pens; 20 heifers/pen) with a stocking density of 14 m^2^/heifer (**HDENS-XL**), or (c) 1 of 6 pastures (1.38-ha pastures; 10 heifers/pasture) with a stocking density of 1,380 m^2^/heifer (**CON**). The HDENS and CON heifers (120 heifers total) were the same animals used by Prado et al. (2026), with drylot pens and pastures (6 pens and 6 pastures) serving as the experimental units for the current experiment. Pastures were regularly mowed to ensure negligible forage availability for CON heifers.

All heifers received the same limit-fed total mixed ration (**TMR**) which averaged 9 kg/heifer daily (dry matter basis) and had free-choice access to water during the experimental period (d 0 to 170; Table 1). The TMR was offered at 0800 h daily in feed bunks with similar linear space across treatments (0.72 m/heifer), such that HDENS-XL pens had double the total bunk space compared with HDENS pens and CON pastures. The TMR was consumed within 6 h of feeding.

**Table 1:** Composition and nutritional profile of the total mixed ration offered to heifers during the experiment.

Item	Component
**Composition, dry matter basis**	
** Corn silage, %**	44.0
** Sorghum sudan hay, %**	28.0
** Cracked corn, %**	12.0
** Dried distillers’ grain**	9.00
** Liquid molasses, %**	5.00
** Mineral mix,^a^ %**	2.00
**Nutritional profile,^b^ dry matter basis**	
** Net energy for maintenance, Mcal/kg**	1.56
** Net energy for gain, Mcal/kg**	0.97
** Total digestible nutrients, %**	68.0
** Neutral detergent fiber, %**	41.3
** Crude protein, %**	13.2

a Containing 21% Ca, 0.01% P, 21% NaCl, 0.20% K, 0.10% Mg, 0.045% Cu, 0.001% Se, 0.280% Zn, 220,000 IU/kg of vitamin A, 19,800 IU/kg of vitamin D3, and 3500 IU/kg of vitamin E (Anipro Xtraperformance Feeds, College Station, TX), as well as sodium monensin (Rumensin; Elanco Animal Health, Greenfield, IN) at 1320 g/ton (37.2 g/ton of total mixed ration, dry matter basis).

b Based on wet chemistry procedures by a commercial laboratory (Dairy One Forage Laboratory, Ithaca, NY). Calculations for net energy for maintenance and gain used the equations proposed by the NRC (2000).

### Sampling

The same sampling schedule from Prado et al. (2026) was adopted in this experiment. Briefly, heifer shrunk BW was recorded after 16 h of feed and water withdrawal on d -3 and 171 to represent initial and final BW, respectively, and used to calculate average daily gain (**ADG**). Heifer temperament was assessed via chute score and exit velocity on d 0, 86, and 170 (Cooke 2014). Heifers were fitted with an ear tag (CowManager, Select Sires, Plain City, OH) on d 0 to record behavioral responses (Wolfger et al. 2015; Pereira et al. 2018). Heifer full BW and blood samples were collected weekly during the experiment (d 0 to 170). Blood was collected via jugular venipuncture into sodium-heparin tubes (Vacutainer, 6 mL; Becton Dickinson, Franklin Lakes, NJ) for plasma collection. CowManager data from sampling days were excluded to avoid confounding effects of handling. Growth rate of each heifer was modeled by linear regression of full BW against sampling days, and each regression coefficient was used as individual growth response. Heifers were allowed to rest for 1 h with access to water and shade prior to sampling (Harvey et al. 2024). Additional blood samples were collected on d 0, 58, 114, and 170 for whole-blood mRNA analysis (Pohler et al. 2017). Hair samples were collected from the tail switch on d 0, 30, 58, 85, 114, 142, and 170 for cortisol analysis, as described by Schubach et al. (2017).

### Laboratorial analyses

All procedures were conducted using the same procedures described by Prado et al. (2026). All blood samples were placed on ice immediately after collection, centrifuged (2500 × g for 30 min at 4°C), and stored at −80°C on the day of collection. Plasma samples collected weekly were analyzed for progesterone concentrations using a commercial radioimmunoassay kit (radioimmunoassay kit #07–170105, MP Biomedicals, Santa Ana, CA), as in Pohler et al. (2016). Heifers were considered pubertal when plasma progesterone concentrations were ≥ 1.5 ng/mL followed by a cyclic pattern indicative of normal estrous cycles (Cooke and Arthington 2009). Heifer age and BW at puberty were determined based on weekly BW measurements and age at the week of puberty attainment. Cortisol was extracted from hair samples as in Schubach et al. (2017), using an enzyme-linked immunoassay cortisol kit (Salimetrics Expanded Range, High Sensitivity 1-E3002, State College, PA). The intra- and inter-assay CV were, respectively, 6.09 and 3.44% for progesterone and 5.12 and 5.77% for cortisol.

For whole-blood mRNA analysis, white blood cells were isolated via buffy coat extraction (Pohler et al. 2017), preserved in 1 mL of TRIzol reagent (Invitrogen, Carlsbad, CA), and stored at −80°C. Total RNA extraction, assessment of RNA quantity and quality, reverse transcription, and real-time polymerase chain reaction (**PCR**) were performed as described by Rodrigues et al. (2015). Responses from the genes of interest were quantified based on the threshold cycle (**CT**), the number of PCR cycles required for target amplification to reach a predetermined threshold. The CT responses from genes of interest were normalized by the geometrical mean of CT values of *ribosomal protein 9* and *β-actin* (Vandesompele et al. 2002). The CV for the geometrical mean of reference genes across all samples was 3.75%. Results are expressed as relative fold change (2^ΔΔ^CT; Ocón-Grove et al. 2008).

### Statistical analysis

All data were analyzed using drylot pen or pasture (6 replications/treatment) as the experimental unit, and replication (treatment × year), heifer(replication), and year as random variables. Quantitative data were analyzed using the MIXED procedure of SAS (SAS Inst. Inc., Cary, NC), whereas binary data were analyzed using the GLIMMIX procedure of SAS. All data were analyzed using the Satterthwaite approximation to determine the denominator degrees of freedom for tests of fixed effects. Model statements used for heifer ADG, initial and final BW, growth rate, temperament variables, and BW and age at puberty contained the effects of treatment. Model statements for puberty attainment, physical activity, and physiological variables contained the effects of treatment, day, and the treatment × day interaction. Blood and hair variables were analyzed using the result from d 0 as an independent covariate. The specified term for the repeated statement was day, the subject was heifer(replication), and the covariance structure utilized was autoregressive, which provided the best fit for these analyses according to the Akaike information criterion. All results are reported as least square means, or covariately adjusted least square means for blood and hair variables, and separated using PDIFF adjusted to the Tukey–Kramer method to prevent Type I errors. Significance was set at *p* ≤ 0.05 and tendencies were determined if *p* > 0.05 and ≤ 0.10. Results are reported according to the effect of treatment if no interactions were significant, or according to the highest-order interaction detected.

## Results and discussion

Results from the present study and its companion manuscript (Prado et al. 2026) complement previous research evaluating the effects of high stocking density on replacement beef heifers reared in drylot systems (Schubach et al. 2017; Harvey et al. 2024). Prado et al. (2026) reported that reducing stocking density attenuated stress-related physiological responses, but failed to prevent delayed puberty attainment in drylot heifers. Alternatively, research from other intensively reared livestock species has demonstrated that space configuration and group size also influence physical activity, space use, social interactions, and stress responses independent of stocking density (Estevez et al. 2007). Increasing pen size at a constant stocking density improved physical activity and space utilization in poultry (Leone and Estevez 2008), dairy cattle (Telezhenko et al. 2012), and veal calves (Abdelfattah et al. 2013). Larger group sizes have also been associated with increased locomotion and altered social dynamics in cattle (Takeda et al. 2003; Færevik et al. 2007) and other species (Estevez et al. 2003; Andersen et al. 2004). Accordingly, this experiment evaluated whether increasing drylot dimensions and group size, while maintaining a constant high stocking density (14 m^2^/heifer), improves behavioral, physiological, and reproductive development of beef heifers reared in drylots. Differences between CON and HDENS heifers are not discussed in detail as these were described in the companion manuscript (Prado et al. 2026). Treatment effects herein should be interpreted as the combined influence of spatial configuration and group size, as the present study did not investigate the independent effects of either factor independently.

### Heifer physical activity and growth responses

Heifer age on day 0 and initial shrunk BW are reported in Table 2 and did not differ (*p* ≥ 0.93) among treatments. Physical activity variables recorded using the CowManager system are reported in Table 2, and treatment effects were detected (*p* ≤ 0.05) for time spent eating, non-active, active, highly active and ruminating. Time spent eating was greater (*p* < 0.01) in CON compared with HDENS and HDENS-XL heifers. Time spent non-active, active and highly active were less (*p* < 0.01) in CON heifers compared with HDENS and HDENS-XL heifers, whereas time spent active was also less (*p* = 0.04) in HDENS compared with HDENS-XL heifers. Time spent ruminating was less (*p* ≤ 0.05) in HDENS-XL compared with CON and HDENS heifers. No other treatment differences were noted (*p* ≥ 0.51) for physical activity variables.

**Table 2:** Growth responses and physical activity in replacement beef heifers reared in paddocks with 10 heifers each (1380 m^2^/heifer; **CON**, *n* = 6), or in drylots with high stocking density (14 m^2^/heifer) and 10 heifers (**HDENS**, 14 × 10 m; *n* = 6) or 20 heifers (**HDENS-XL**, 28 × 10 m; *n* = 6) per drylot for 170 d.^1^

Item	CON	HDENS	HDENS-XL	SEM	*P =*
**Age (d 0), d**	271	271	270	2	0.93
**Physical activity,** **Time spent,^2^ %**					
** Active**	8.79^c^	10.8^b^	12.2^a^	0.5	< 0.01
** Eating**	21.0^a^	14.1^b^	13.7^b^	0.7	0.02
** Highly active**	18.0^b^	20.0^a^	20.4^a^	0.4	< 0.01
** Non-active**	25.2^b^	27.6^a^	28.0^a^	0.4	< 0.01
** Ruminating**	27.0^a^	27.4^a^	25.6^b^	0.5	0.02
**Growth responses^3^**					
** Initial shrunk BW (d -3), kg**	232	232	232	2	0.99
** Final shrunk BW (d 171), kg**	345	347	339	8	0.76
** Average daily gain, kg/d**	0.641	0.655	0.611	0.026	0.67

1 Within rows, values with different superscripts differ (*p* ≤ 0.05).

2 Activity parameters were evaluated as in Wolfger et al. (2015) according to the CowManager system (Select Sires, Plain City, OH). Results are reported according to % of the day performing each activity.

3 Calculated using initial (d -3) and final (d 171) shrunk BW, which was recorded after 16 h of feed and water withdrawal.

Time spent eating was greater in CON heifers compared with drylot treatments as in Prado et al. (2026), which can be attributed to slight forage regrowth that elicited grazing-related head and neck movements classified as time spent eating and not as locomotor activity (Wolfger et al. 2015; Pereira et al. 2018). This rationale corroborates the reduced time spent non-active, active, and highly active in CON heifers compared with HDENS and HDENS-XL, which likely resulted at the expense of time spent eating. The CowManager system in Prado et al. (2026), however, primarily distinguished behavioral differences between pasture and drylot heifers, and not among drylot heifers reared in different stocking densities. In the present experiment, time spent active and ruminating also differed between HDENS and HDENS-XL heifers. These differences are consistent with increased space availability and group size promoting greater physical activity and space use (Leone and Estevez 2008; Telezhenko et al. 2012; Abdelfattah et al. 2013). Rumination is primarily driven by diet composition and intake pattern rather than housing conditions (Grant et al. 1990; Beauchemin 1991); however, redistribution of daily time budgets toward increased physical activity and social behaviors may reduce time available for ruminating (Munksgaard and Simonsen 1996; Jensen 2004). Hence, the reduced ruminating time observed in HDENS-XL heifers compared with CON and HDENS heifers likely reflects behavioral redistribution rather than altered nutritional status or impaired rumen function. Therefore, modifying drylot dimension and group size under a constant high stocking density altered behavioral time allocation of heifers reared in drylots, corroborating that space configuration influences physical activity patterns independent of stocking density (Estevez et al. 2007).

No treatment effects were detected (*p* ≥ 0.67) for heifer ADG and final shrunk BW (Table 2). The growth rate of each heifer, modeled using linear regression of full BW and sampling days, also did not differ (*p* = 0.48) among treatments (0.767, 0.759, and 0.727 kg/d for CON, HDENS, and HDENS-XL, respectively; SEM = 0.026). These outcomes corroborate the similar nutritional management imposed across treatments, as reported in the companion study (Prado et al. 2026) and previous studies from our group (Schubach et al. 2017; Harvey et al. 2024). Others have reported greater ADG in drylot heifers compared with pasture-reared cohorts and attributed these differences to differences in physical activity; however, those responses were likely driven by differences in dietary management as drylot heifers received a TMR (Petersen et al. 2014; Perry et al. 2015). Schubach et al. (2017) suggested that altered physical activity may impact energy requirements for maintenance, despite reporting similar ADG in drylot- and pasture-reared heifers. The same rationale can be applied herein, given that ADG did not differ among treatments despite the differences observed for physical activity. Hence, results from this experiment and the companion manuscript (Prado et al. 2026) indicate that heifer growth rate was unaffected by modifications in drylot configuration and group size, as well as differences in stocking density when nutritional management was similar across treatments.

### Physiological parameters

#### Hair cortisol

**Figure 1:**
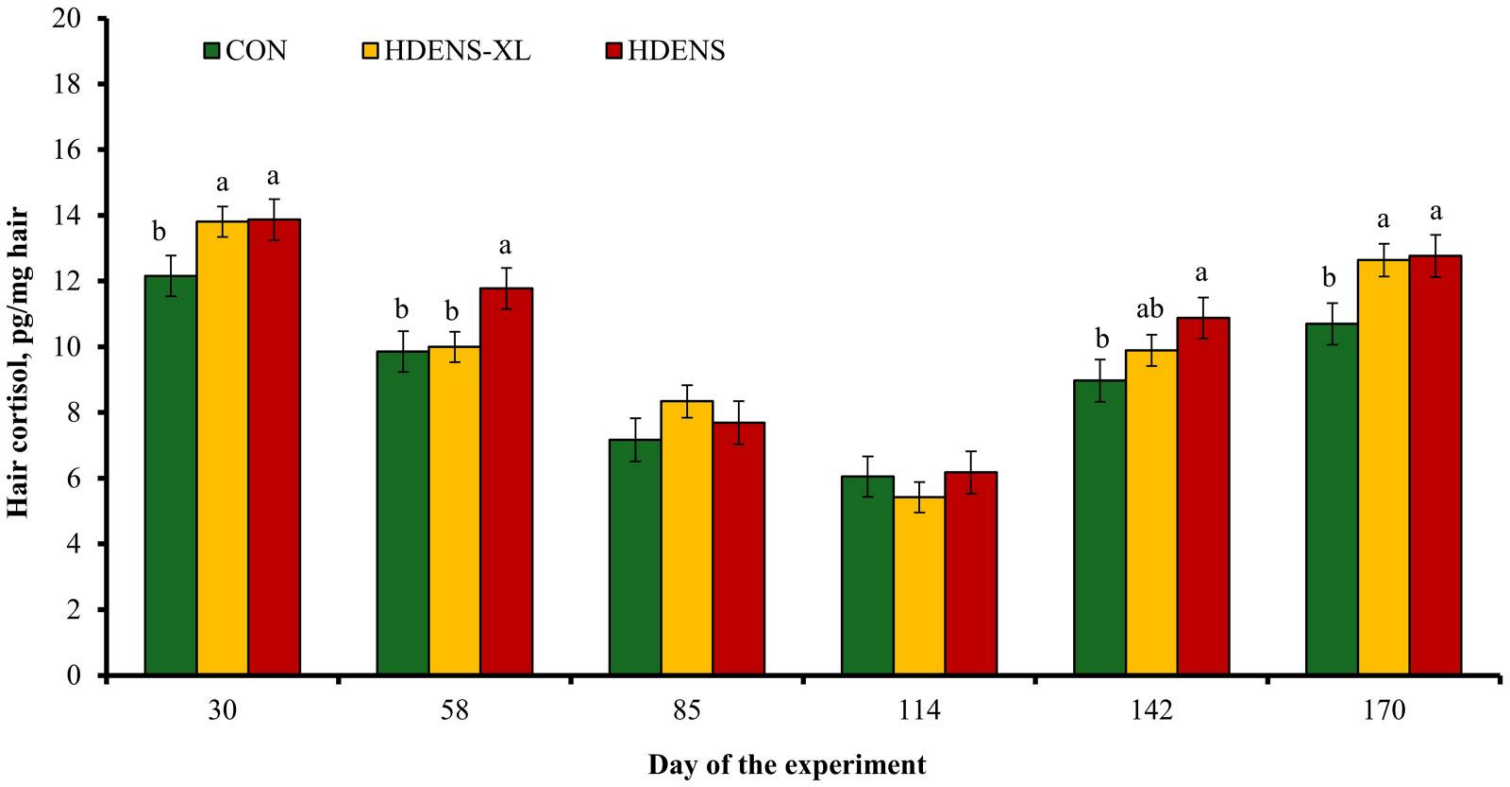
Hair cortisol concentrations in replacement beef heifers reared in paddocks with 10 heifers each (1380 m^2^/heifer; **CON**, *n* = 6), or in drylots with high stocking density (14 m^2^/heifer) and 10 heifers (**HDENS**, 14 × 10 m; *n* = 6) or 20 heifers (**HDENS-XL**, 28 × 10 m; *n* = 6) per drylot for 170 d. Hair samples were collected on d 0, 30, 58, 85, 114, 142, and 170 as in Schubach et al. (2017). results from d 0 were used as independent covariate. A treatment × day interaction was detected (*p* = 0.04). within days, means with different superscripts differ (*p* ≤ 0.05).

A treatment × day interaction was detected (*p* = 0.04) for hair cortisol concentrations (Fig. 1), which were greater (*p* ≤ 0.05) for HDENS and HDENS-XL compared with CON heifers on d 30 and 170, greater (*p* ≤ 0.04) for HDENS compared with CON and HDENS-XL heifers on d 58, and greater (*p* = 0.03) for HDENS compared with CON heifers on d 142. No other treatment differences were noted within days (*p* ≥ 0.15). As reported in the companion manuscript (Prado et al. 2026), HDENS heifers experienced greater chronic stress during most of the experimental period compared with CON heifers, given that hair cortisol concentration in tail-switch hair is considered a biomarker of chronic stress in cattle (Moya et al. 2013; Burnett et al. 2014; Cooke et al. 2017). In this experiment, increasing drylot dimensions and group size was associated with reduced hair cortisol concentrations at select time points (d 58); however, this response was not consistent across the experimental period. Prado et al. (2026) concluded that reducing stocking density deferred, but did not prevent the onset of chronic stress in drylot heifers. Hence, drylot housing under high stocking density represents a major contributor to chronic stress, whereas modifications in drylot configuration and group size were insufficient to lessen adrenocortical activity to levels observed in pasture heifers.

#### Whole blood cells, mRNA expression

**Figure 2:**
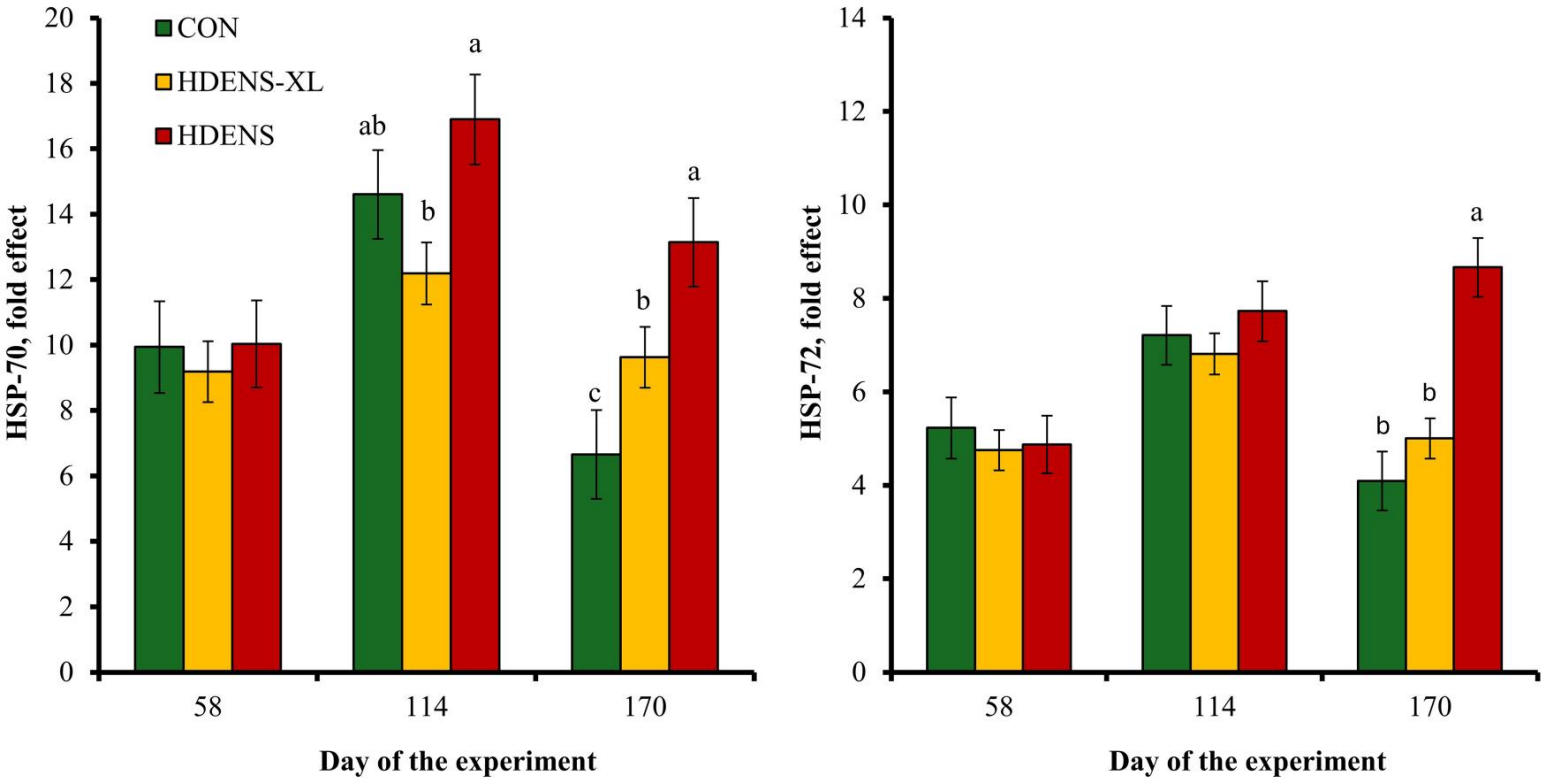
mRNA expression of *heat shock protein 70* (**HSP70**) and *heat shock protein 72* (**HSP72**) in whole blood of replacement beef heifers reared in paddocks with 10 heifers each (1380 m^2^/heifer; **CON**, *n* = 6), or in drylots with high stocking density (14 m^2^/heifer) and 10 heifers (**HDENS**, 14 × 10 m; *n* = 6) or 20 heifers (**HDENS-XL**, 28 × 10 m; *n* = 6) per drylot for 170 d. Samples were collected on d 0, 58, 114, and 170 of the experiment, processed as in Pohler et al. (2017), evaluated for mRNA expression according to Rodrigues et al. (2015), and reported as in Ocón-Grove et al. (2008). Data were analyzed using results from d 0 as independent covariate in each respective analysis. A treatment × day interaction was detected (*p* ≤ 0.03) for both variables. Within days, means with different superscripts differ (*p* ≤ 0.05).

A treatment × day interaction was detected (*p* = 0.03) for blood mRNA expression of *heat shock protein* (**HSP**)-70 (Fig. 2), which was greater (*p* < 0.01) for HDENS compared with HDENS-XL heifers on d 114, and greater (*p* ≤ 0.05) for HDENS compared with HDENS-XL and CON heifers on d 170. Expression of HSP-70 mRNA on d 170 was also greater (*p* = 0.03) for HDENS-XL compared with CON heifers. A treatment × day interaction was also detected (*p* < 0.01) for blood mRNA expression of HSP-72 (Fig. 2), which was greater (*p* < 0.01) for HDENS compared with HDENS-XL and CON heifers on d 170. No other treatment differences were detected (*p* ≥ 0.14) for mRNA expression of HSP-70 and HSP-72 during the experiment.

As described by Prado et al. (2026), HDENS heifers experienced heightened cellular stress toward the end of the experimental period compared with CON heifers, as mRNA expression of HSP in whole blood is recognized as biomarker of cellular stress (Welch 1992). Increasing drylot dimension and group size herein attenuated this cellular stress response, as evidenced by HSP-70 mRNA expression on d 114 and HSP-72 mRNA expression on d 170 being comparable to values observed in pasture heifers. Results from Prado et al. (2026) also demonstrated that reducing stocking density to 28 or 42 m^2^/heifer mitigated cellular stress responses associated with prolonged drylot housing. Collectively, cellular stress response to drylot housing can be partially attenuated by increasing drylot dimension and group size (Estevez et al. 2007), as well as by decreasing stocking density as reported in the companion manuscript (Prado et al. 2026).

### Temperament responses

Heifer temperament responses are reported in Table 3, whereas no treatment effects were detected (*p* ≥ 0.20) for chute score, exit velocity, and temperament score. No treatment × day interactions were detected for these temperament variables (*p* ≥ 0.25). Hence, increasing pen dimension and group size to heifers reared in high stocking density did not impact chute-based temperament responses herein. Harvey et al. (2024) reported that exit velocity and temperament score were less in pasture heifers compared with drylot heifers. The 170-d duration of this experiment may have been insufficient to elicit detectable differences in temperament among treatments (Prado et al. 2026). Temperament score reflects behavioral responses to a specific situation at a single point in time, and does not independently characterize the animal’s affective or welfare state (Lee et al. 2018; Hubbard et al. 2019). As in the companion manuscript, physiological indicators of chronic stress were more responsive than chute-based behavioral measures in detecting stress-related effects of stocking density herein (Prado et al. 2026).

**Table 3:** Temperament and puberty responses in replacement beef heifers reared in paddocks with 10 heifers each (1380 m^2^/heifer; **CON**, *n* = 6), or in drylots with high stocking density (14 m^2^/heifer) and 10 heifers (**HDENS**, 14 × 10 m; *n* = 6) or 20 heifers (**HDENS-XL**, 28 × 10 m; *n* = 6) per drylot for 170 d.^1^

Item	CON	HDENS	HDENS-XL	SEM	*P =*
**Temperament variables^2^**					
** Chute score**	1.36	1.35	1.33	0.07	0.95
** Exit velocity (m/s)**	2.61	2.43	2.56	0.08	0.42
** Temperament score**	2.23	2.00	2.17	0.08	0.20
**Final puberty attainment,^3^%**	73.3	61.0	66.7	5.5	0.36
** Age at puberty, d**	370^b^	399^a^	375^b^	5	< 0.01
** Body weight at puberty, kg**	311	331	313	8	0.19

1 Within rows, values with different superscripts differ (*p* ≤ 0.05).

2 According to the techniques described by Cooke (2014). Evaluated on d 0, 86, and 170 of the experiment. Results from d 0 were used as independent covariate in each respective analysis. No treatment × day interactions were detected for temperament variables (*p* ≥ 0.25).

3 Evaluated according to plasma progesterone concentrations in samples collected every 7 d from d 0 to 170 (Cooke and Arthington 2009).

### Puberty attainment

**Figure 3:**
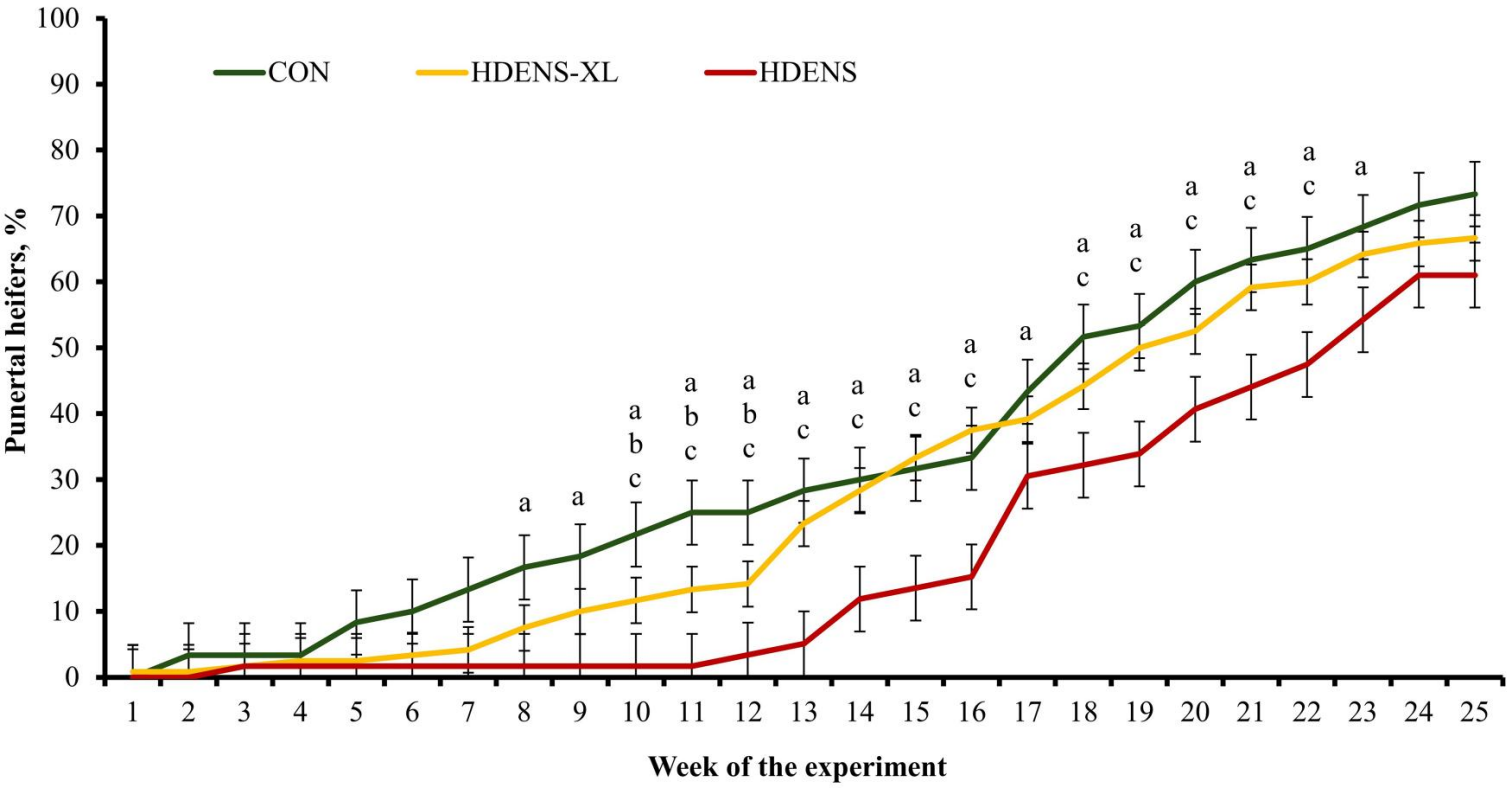
Puberty attainment in replacement beef heifers reared in paddocks with 10 heifers each (1380 m^2^/heifer; **CON**, *n* = 6), or in drylots with high stocking density (14 m^2^/heifer) and 10 heifers (**HDENS**, 14 × 10 m; *n* = 6) or 20 heifers (**HDENS-XL**, 28 × 10 m; *n* = 6) per drylot for 170 d in. Puberty was evaluated according to plasma progesterone concentrations in samples collected weekly from d 0 to 170. Heifers were considered pubertal once plasma progesterone concentrations were ≥ 1.5 ng/mL followed by a cyclic pattern of plasma progesterone and < and ≥ 1.5 ng/mL suggestive of normal estrous cycles (Cooke and Arthington 2009). A treatment × day interaction was detected (*p* < 0.01). Within weeks, superscripts indicate treatment differences (*p* ≤ 0.05) between: a = CON vs HDENS, b = CON vs. HDENS-XL, c = HDENS vs. HDENS-XL.

A treatment × day interaction was detected (*p* < 0.01) for puberty attainment (Fig. 3). Puberty rate was greater (*p* ≤ 0.05) in CON compared with HDENS heifers on weeks 8, 9, 17, and 23. From weeks 10 to 12, puberty rate was greater (*p* ≤ 0.05) in CON compared with HDENS and HDENS-XL heifers. From weeks 13 to 16, and from 18 to 22, puberty rate was greater (*p* ≤ 0.05) in CON and HDENS-XL compared with HDENS heifers. No other treatment differences were noted within weeks for puberty attainment (*p* ≥ 0.13). Final puberty attainment and BW at puberty did not differ (*p* ≥ 0.19) among treatments (Table 3); however, age at puberty was less (*p* < 0.01) in CON and HDENS-XL compared with HDENS heifers, and did not differ (*p* = 0.47) between CON and HDENS-XL heifers.

As described by Prado et al. (2026) and our previous studies (Schubach et al. 2017; Harvey et al. 2024) heifers reared in drylots with stocking density of 14 m^2^/heifer (10 heifers/drylot) had delayed puberty attainment compared with heifers reared on pasture. This delay in puberty attainment was associated with greater chronic stress experienced by drylot heifers (Dobson and Smith 2000) and observed despite similar BW gain among treatments, which is a major regulator of age at puberty in cattle (Schillo et al. 1992). Supporting our hypothesis, increasing drylot dimension and group size attenuated the puberty delay in drylot heifers reared at 14 m^2^/heifer. These outcomes can be associated with equivalent levels of HSPs mRNA expression in whole blood and, to some extent, equivalent hair cortisol concentrations between CON and HDENS-XL heifers during specific time points within the experiment. The increase in physical activity of HDENS-XL heifers may also have contributed to their hastened puberty compared with HDENS heifers as suggested by our previous studies (Schubach et al. 2017; Harvey et al. 2024). Exercise modulates circulating concentrations of endogenous opioids (Harber and Sutton 1984) which promote secretion of gonadotropins (Mahmoud et al. 1989). Therefore, increasing drylot dimension and group size to heifers reared at high stocking density attenuated physiological stress responses, resulting in puberty attainment comparable to heifers reared on pasture.

### Conclusions

As described in the companion manuscript (Prado et al. 2026), beef heifers reared in drylots with a high stocking density (14 m^2^/heifer; HDENS) experienced heightened stress-related physiological responses and delayed puberty attainment compared to heifers reared on 1.38-ha pastures. Increasing the dimension and the number of heifers within each drylot pen, but maintaining the stocking density at 14 m^2^/heifer improved heifer physical activity and lessened stress responses by the end of the experimental period, which resulted in similar puberty achievement compared with pasture-reared controls. Hence, drylot dimensions and group size influence and should be considered to foster welfare and reproductive development of beef heifers reared in intensive drylot systems. Results from this experiment and Prado et al. (2026) are novel and provide foundational data for optimal management and stocking densities for heifers reared in drylots.

## List of abbreviations

ADG: average daily gain
BW: body weight
CT: threshold cycle
HSP: heat shock protein
TMR: total mixed ration
PCR: polymerase chain reaction
